# Dose optimization in surgical prophylaxis: sub-inhibitory dosing of vancomycin increases rates of biofilm formation and the rates of surgical site infection

**DOI:** 10.1038/s41598-023-30951-y

**Published:** 2023-03-21

**Authors:** Kimberly M. Brothers, Dana M. Parker, Masashi Taguchi, Dongzhu Ma, Jonathan B. Mandell, Lance L. Thurlow, Venkata C. Byrapogu, Kenneth L. Urish

**Affiliations:** 1 Arthritis and Arthroplasty Design Group, Department of Orthopaedic Surgery, University of Pittsburgh, Pittsburgh, PA USA; 2 Department of Orthopaedic Surgery, Ageo Medical Clinic, 3133 Haraichi, Ageo-Shi, Saitama, Japan; 3 Department of Microbiology and Immunology, University of North Carolina, Chapel Hill, NC USA; 4 Arthritis and Arthroplasty Design Group, and The Bone and Joint Center, Department of Orthopaedic Surgery, Department of Bioengineering, Department of Biomedical Engineering, and Clinical and Translational Science Institute, Magee Womens Hospital of the University of Pittsburgh Medical Center, University of Pittsburgh, Pittsburgh, PA 15219 USA

**Keywords:** Microbiology, Biofilms, Risk factors

## Abstract

Antibiotic stewardship is viewed as having great public health benefit with limited direct benefit to the patient at the time of administration. The objective of our study was to determine if inappropriate administration of antibiotics could create conditions that would increase the rates of surgical infection. We hypothesized that sub-MIC levels of vancomycin would increase *Staphylococcus aureus* growth, biofilm formation, and rates of infection. *S. aureus* MRSA and MSSA strains were used for all experiments. Bacteria were grown planktonically and monitored using spectrophotometry. Quantitative agar culture was used to measure planktonic and biofilm bacterial burden. A mouse abscess model was used to confirm phenotypes in vivo. In the planktonic growth assay, increases in bacterial burden at ¼ MIC vancomycin were observed in USA300 JE2 by 72 h. Similar findings were observed with ½ MIC in Newman and SH1000. For biofilm formation, USA300 JE2 at ¼ and ½ MIC vancomycin increased biofilm formation by approximately 1.3- and 2.3-fold respectively at 72 h as compared to untreated controls. Similar findings were observed with Newman and SH1000 with a 2.4-fold increase in biofilm formation at ½ MIC vancomycin. In a mouse abscess model, there was a 1.2-fold increase with sub-MIC vancomycin at 3 days post infection. Our study showed that Sub-optimal vancomycin dosing promoted *S. aureus* planktonic growth and biofilm formation, phenotypic measures of bacterial virulence. This phenotype induced by sub-MIC levels of vancomycin was also observed to increase rates of infection and pathogenesis in our mouse model. Risks of exposure to sub-MIC concentrations with vancomycin in surgical procedures are greater as there is decreased bioavailability in tissue in comparison to other antibiotics. This highlights the importance of proper antibiotic selection, stewardship, and dosing for both surgical prophylaxis and treatment of infection.

## Introduction

Antibiotic stewardship is an important strategy to prevent development of antibiotic resistant organisms^1^. This is the primary motivational force behind the appropriate and judicious use of surgical antibiotic prophylaxis^2,3^, and limiting non-essential post-operative use of antibiotics in orthopaedic surgery. This has great public health benefit. At an individual level, implementing strict antibiotic stewardship has a less obvious benefit. Surgical site infections (SSIs) and implant associated infections can have severe outcomes with a high morbidity, mortality, and a large economic burden^4–8^. This is in comparison to the adverse events associated with antibiotics including allergic reaction, organ injury, and medication intolerance^9^. When clinical evidence is unclear, the potential benefits of antibiotic use are perceived to outweigh the possible adverse events. This has fuelled the debate between the use of a single dose of antibiotics as surgical prophylaxis^10,11^ and extended antibiotic treatments to prevent or treat periprosthetic joint infection^12–14^. Antibiotic stewardship is not just proper antibiotic selection but also obtaining appropriate clinical therapeutic levels.

Proper antibiotic therapeutic levels are a critical component for appropriate surgical prophylaxis which includes dose and length of administration as determined by the area under the curve. At levels below the minimum inhibitory concentration (MIC), antibiotics have been observed to alter growth rates of many bacteria including *Staphylococcus aureus*, the primary organism associated with orthopaedic infection^15–23^. This is a particular concern as bone and joints are less permeable to antibiotics and have a notoriously lower level of bioavailability as compared to other tissue leading to exposure to sub-MICs of antibiotics^24^. This problem is further compounded by the presence of an antibiotic tolerant biofilm on prosthetic components and within the joint space. Compared to their planktonic state, bacteria in a biofilm are 1000 times more resistant to antibiotics^25–27^.

The focus of this study was to determine the effect of sub-MIC levels of vancomycin on growth and pathogenesis of *S. aureus*, the most common organism associated with SSIs. We hypothesized that low doses of vancomycin would increase rates of infection due to increases in bacterial growth and biofilm formation. Our results demonstrate that sub-MIC levels of vancomycin increased *S. aureus* planktonic growth rates and biofilm formation in vitro and was associated with increased virulence in vivo.

## Methods

### Bacterial strains, plasmids and growth conditions

*Staphylococcus aureus* MRSA (USA300 JE2) was purchased from The American Type Culture Collection (ATCC). MSSA (SH1000, Newman) and EGFP reporter plasmids were kindly provided by Dr. Niles Donegan and Dr. Ambrose Cheung. *S. aureus* strains were grown in trypticase soy broth (TSB) medium with shaking at 37 °C and stored as frozen stocks in 15% glycerol at – 80 °C.

### Minimum inhibitory concentration (MIC) assay

All vancomycin and cefazolin (Sigma-Aldrich) minimum inhibitory concentrations (MIC) for *S. aureus* JE2, Newman and SH1000 were determined according to CLSI protocols^28,29^. Bacteria were inoculated into 5 ml TSB medium and grown overnight at 37ºC. Cultures were normalized to 1 × 10^6^ CFU/ml using a 0.5× McFarland turbidity standard (Hardy Diagnostics) and plated in 96 well plates (Costar) containing serial dilutions of vancomycin (0.25, 0.5, 1, 2, 4, 8, 16, 32, 64 µg/ml). As an additional measure for MIC *S. aureus* MRSA JE2 and MSSA Newman containing an enhanced green fluorescent protein (EGFP) were tested with vancomycin E-test MIC strips (Liofilchem). A culture sample (~ 10^8^ CFU/ml) was obtained and streaked onto a trypticase soy blood agar plate (TSA II). Next, the E-test MIC strip was applied and the plate was incubated overnight at 37 °C. MIC values were recorded where the pointed end of the inhibition ellipse intersects with the side of the strip. Newman was visualized using a Nikon TE-300 fluorescent microscope equipped with a SPOT camera.

### Planktonic MIC growth assay

Approximately 1 × 10^4^ CFU MRSA JE2, MSSA Newman and SH1000 were added to a 12-well plate (Costar) containing 0, 0.25, and 0.5 μg/ml vancomycin respectively (0, ¼ and ½ MIC) and incubated at 37 °C for 24, 48, and 72 h. Bacteria were serially diluted, plated onto TSA II plates, and incubated overnight at 37 °C. Colonies were counted and the bacterial concentration was determined in CFU/ml. To compare phenotypes, MSSA strains Newman and SH1000 were tested with 0, 0.0125 and 0.25 μg/ml cefazolin (0, ¼ and ½ MIC).

### Titanium rod biofilm MIC assay

Titanium rods (12 mm) were placed into a 12 well tissue culture plate containing TSB with 1 × 10^4^ CFU/ml *S. aureus* MRSA (JE2), MSSA Newman and SH1000 with 0, 0.25, and 0.5 μg/ml vancomycin respectively (0, ¼ and ½ MIC). The plate was incubated at 37 °C for 24, 48, and 72 h. To remove non-adherent cells, wells were replaced with fresh TSB every 24 h. At experimental endpoints, titanium rods were washed three times in 1 ml PBS and placed into 1 ml of PBS containing 0.1% tween (PBST). To break up the biofilm, samples were sonicated using a Branson model #3510 sonicating water bath for 30 min. Serial dilutions of the sonicated bacterial suspension were prepared and plated onto TSA II plates and incubated overnight at 37 °C. Colonies were counted and the biofilm bacterial burden was determined in CFU/ml.

### Fibrinogen biofilm assay

A semi-quantitative adherence assay was performed with MRSA JE2 using 96-well plates. Plates were coated with 100 μl of phosphate-buffered saline (PBS) containing 5 μg/ml fibrinogen (Sigma-Aldrich) and incubated overnight at 4 °C. Plates were washed three times with PBS and blocked with 2% bovine serum albumin (BSA) (Sigma-Aldrich) for 1 h at 37 °C. Plates were washed with PBS as described above. Approximately 1 × 10^7^ bacteria were added to appropriate wells and incubated for 24 h at 37 °C. Next, wells were washed four times with PBS. Bacteria were fixed in 10% formaldehyde (Sigma-Aldrich) for 10 min. 0.2% crystal violet (Sigma-Aldrich) was added to each well and incubated at room temperature for 10 min. The plates were washed four times with distilled water and air dried for 2 h. Crystal violet stain was dissolved in 30% acetic acid (Fisher Scientific) and absorbance was measured at 590 nm using an Infinite 200 plate reader (Tecan).

### Mouse abscess model

8–12-week-old C57BL/6J female mice were purchased from The Jackson laboratory (Bar Harbor, ME). All animal protocols used for these experiments were approved by the University of Pittsburgh’s Institutional Animal Care and Use Committee. Authors complied with all the relevant guidelines and regulations and complied with ARRIVE guidelines. The weight of each C57BL/6J mouse was recorded before the start of the procedure. One hour prior to inoculation of bacteria, 0, 0.01, and 0.1 mg/g vancomycin were injected subcutaneously into mice (n = 10 per group). The in vitro and in vivo doses are related as they are below the MIC and sub-therapeutic. There is not a one-to-one correlation between in vitro and in vivo doses based on antibiotic pharmacokinetics and bioavailability. Mice were anesthetized by 2% isoflurane and the hair on the flank was removed using Nair. Next, 20 µl of *S. aureus* (1 × 10^6^ CFU) was injected subcutaneously into the flank. At 3 days post infection, mice were euthanized and the length and width of the abscess was measured with a Vernier caliper. The abscess area was calculated using the formula Area = (L/2) × (W/2) × π. Each abscess was collected and placed in sterile PBS. Tissues were mechanically homogenized with a TT10 basic Ultra-TURRAX homogenizer (IKA). Next, samples were serially diluted, plated onto TSA II plates and incubated overnight at 37 °C. Colonies were counted and the bacterial burden in CFU/ml was recorded.

### Infection rate and median infectious dose (ID50)

Bacterial cultures and mice were infected and treated as described above. Following the protocol described above for inoculation, serial dilutions of MRSA JE2 were injected into the flank of mice (n = 10 per group). On the 3rd day of infection, animals were euthanized, abscess were measured, harvested, and bacterial burden quantified. The number of bacteria required to infect 50% of the mice (ID50) was calculated according to Reed et al.^30^.

### Statistical analysis

All graphical and statistical analysis was performed using Prism 9.0 (GraphPad, La Jolla, CA). Statistical analysis was performed using two-way ANOVA for the planktonic and biofilm data, and a two-tailed Student’s *t*-test for the in vivo infection data. Significance was determined at p < 0.05.

## Results

### Sub-MIC levels of vancomycin promote *S. aureus* growth

**Figure 1:**
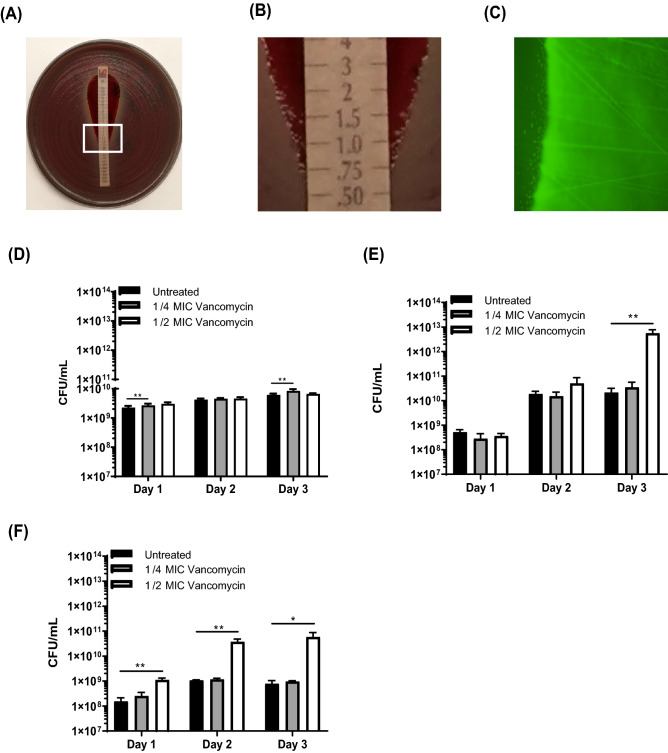
Sub-MIC vancomycin promotes *S. aureus* planktonic growth (**A**) MRSA JE2 vancomycin E-test strip MIC assay. White box indicates zoomed in area for (**B**) a greater bacteria accumulation occurs near the edge of E-test strip inhibitory area (**C**) MSSA Newman strain containing an EGFP fluorescent reporter demonstrating a brighter green fluorescent signal near the edge of the vancomycin inhibitory are a indicating more bacterial growth. Sub-MIC vancomycin promotes increased planktonic growth in (**D**) MRSA JE2 (**E**) MSSA Newman and (**F**) SH1000. N = 4 per treatment group. Error bars represent standard deviation. *p < 0.05, **p < 0.01.

**Figure 2:**
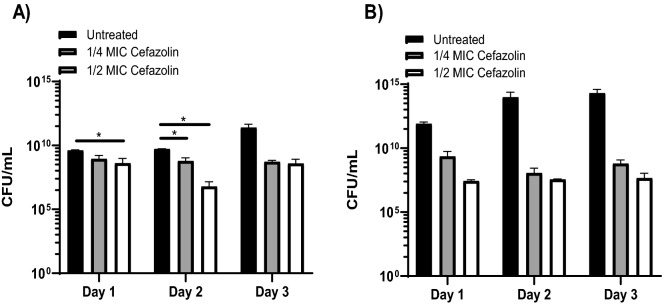
Sub-MIC cefazolin does not promote *S. aureus* planktonic growth. Sub-MIC cefazolin results in decreased planktonic growth in (**A**) MSSA Newman and (**B**) SH1000. *p < 0.05.

When testing MRSA JE2 with a vancomycin E-test strip, we observed increased bacterial growth along the boundary of the growth inhibition area (Fig. 1A, white box). A greater accumulation of *S. aureus* colonies were observed near the edge of inhibitory area at sub-MIC concentrations (Fig. 1B). When this assay was repeated using a *S. aureus* MSSA Newman strain containing an EGFP fluorescent reporter, a brighter green fluorescent signal was observed near the inhibitory edge of the test strip at sub-MIC ranges indicating increased bacterial growth (Fig. 1C). To expand upon these findings, we used a planktonic growth assay. Similar to the E-test results, with JE2 strain, ¼ MIC vancomycin treatment resulted in a 1.2-fold increase after 24 h of growth (p = 0.02) and a 1.1-fold increase after 72 h of growth (0.03). In the Newman strain, ½ MIC vancomycin treatment resulted a 259-fold increase after 72 h growth (p = 0.01). SH1000 phenotypes were similar with ½ MIC vancomycin treatment resulting in a 7.2-fold increase (p = 0.01) after 24 h growth, a 35-fold increase after 48 h growth (p = 0.02) and a 75-fold increase after 72 h growth (p = 0.01). When sub-MIC cefazolin was tested with Newman and SH1000 strains for comparison, the same trend was not observed. Cefazolin treatment at ¼ and ½ MIC levels resulted in decreased bacterial growth in comparison to untreated samples (Fig. 2).

### *S. aureus* treated with sub-MIC concentrations of vancomycin increases biofilm formation

**Figure 3:**
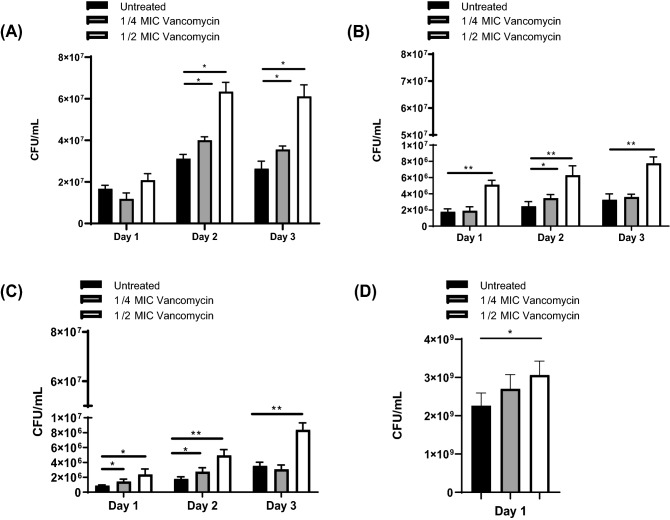
Sub-MIC vancomycin promotes *S. aureus* biofilm growth in (**A**) MRSA JE2, (**B**) MSS A Newman and (**C**) SH1000, (**D**) Sub-MIC vancomycin promotes MRSA JE2 *S. aureus* biofilm growth on fibrinogen coated wells. N = 4 per treatment group. Error bars represent standard deviation. *p < 0.05, **p < 0.01.

After observing increased rates of *S. aureu*s planktonic growth in the presence of sub-MIC levels of vancomycin in all strains tested, we hypothesized a similar phenotype would occur with biofilm formation. In JE2 biofilms, ¼ MIC vancomycin resulted in 1.3-fold increase in bacterial burden on day 2 and day 3 respectively (p = 0.03, p = 0.04, Fig. 2A). JE2 biofilms at ½ MIC vancomycin resulted in 2 and 2.3-fold increases in bacterial burden on day 2 and 3 respectively (p = 0.01p = 0.02 Fig. 3A). Similar results were observed with *S. aureus* Newman at ¼ MIC vancomycin with a 1.4-fold increase in bacterial burden on day 2 (p = 0.03) and ½ MIC vancomycin with 2.9, 2.6, and 2.4-fold increases in bacterial burden on days 1, 2, and 3 (Fig. 3B, p = 0.004, p = 0.002, p = 0.001). SH1000 demonstrated a similar trend with ¼ MIC vancomycin resulting in 1.6 and 1.5-fold increases in bacterial burden on days 1 and 2 (Fig. 3C, p = 0.03, p = 0.02) and ½ MIC vancomycin treatment resulting in 2.7, 2.8, and 2.4-fold increases in bacterial burden on days 1, 2, and 3 (Fig. 3C, p = 0.02, p = 0.001, p = 0.003). Similar to our biofilm results grown on titanium wires, after 1 day of growth, ½ MIC vancomycin treatment on a fibrinogen coated surface resulted in increased biofilm growth with a 1.4-fold increase in comparison to untreated controls (Fig. 3D, p = 0.02).

### Sub-MIC treatment of vancomycin increases *S. aureus* pathogenicity in vivo

**Figure 4:**
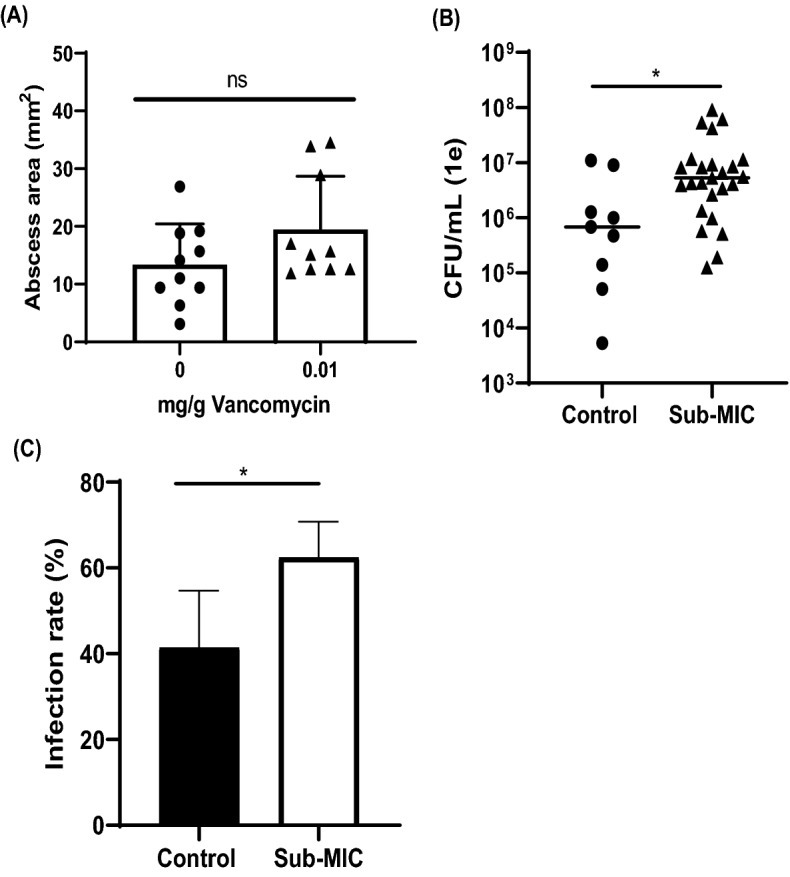
Sub-MIC vancomycin increases bacterial growth and infection in vivo. Mouse abscess model inoculated with 1 × 10^7^ CFU/ml JE2. *S. aureus* and treated with sub-MIC vancomycin (0.01 mg/g) for 3 days. (**A**) There were no differences between treatment groups for total abscess area n = 10 per group. (**B**) For bacterial burden, the abscess burden was significantly greater in the sub-MIC group at 3 days post infection n = 10 per group. (**C**) Sub-MIC vancomycin treatment results in a higher infection rate in mice. Results are from 5 independent experimental trials. Error bars represent standard deviation. *p < 0.05.

To test the phenotype of sub-MIC vancomycin treatment in vivo, we used an SSI abscess mouse model. No differences in abscess volume were observed for any of the treatment groups (Fig. 4A). However, a significantly greater bacterial burden (1.2-fold increase) was observed in mice treated with sub-therapeutic vancomycin dosing in comparison to untreated controls (Fig. 4B, p = 0.009).

### Infection rates are greater in mice treated with sub-MIC concentrations of vancomycin

Rates of bacterial growth and biofilm formation are important phenotypes for pathogenesis. If sub-MIC ranges of vancomycin increased *S. aureus* growth, biofilm formation, and bacterial burden in our mouse model, we hypothesized that treatment with sub-therapeutic levels of vancomycin would result in increased rates of infection. In comparison to untreated controls (41.5% infection rate), a greater infection rate (62.5%) was observed in the sub-therapeutic vancomycin treatment group (Fig. 4C, p = 0.03, 95% CI 4.941 to 37.06).

## Discussion

The clear benefits of antibiotic stewardship at a public health level in preventing multidrug resistant bacteria are not as obvious at the patient level. In the post-operative period, antibiotics have a low risk profile to the patient as compared to the large possible benefit at preventing surgical infection. When the clinical evidence is unclear, the potential benefits of antibiotics from a surgeon perspective are perceived to outweigh the risk of antibiotic overuse and possible adverse events. This has fueled the debate on whether perioperative antibiotics should continue to be administered as surgical prophylaxis, especially with the use of arthroplasty implants^10,11^. Sir John Charnley noted avoiding surgical antibiotic prophylaxis because “it had been suggested that a higher rate of wound infection occurs with prophylactic antibiotics than without^31^”. In 2006, The Centers for Disease Control (CDC) along with the Centers for Medicare & Medicaid Services created the Surgical Care Improvement Project (SCIP) guidelines that recommend (1) prophylactic antibiotics given within 60–120 min prior to surgery, (2) proper antibiotic selection based on procedures, and (3) no additional prophylactic antibiotics after closure of the surgical incision^3^.

In the present study, we observed sub-therapeutic levels of vancomycin increased *S. aureus* planktonic growth and biofilm formation in vitro and in vivo, an important step in the initiation of surgical infection. Increased biofilm formation in the presence of sub-MIC antibiotics has been demonstrated in *S. aureus* and other microorganisms known to cause SSIs including coagulase negative Staphylococci, *Staphylococcus epidermidis*, and *Pseudomonas aeruginosa*^15–17,32^. This phenotype was observed in our animal model where sub-therapeutic levels of vancomycin increased bacterial burden and infection rate. Combined, these data have important implications for surgical prophylaxis, proper antibiotic selection, and dosing which includes the actual dose and timing. Our manuscript suggests that not achieving clinical therapeutic levels of antibiotics such as with shorter duration of surgical prophylaxis as recommended by the CDC may potentially increase the risk for surgical infection.

First generation cephalosporins are the preferred choice for antibiotic prophylaxis in orthopaedic surgery^33^. Patients who receive an alternative treatment to cephalosporins have been shown to have an increased risk for developing SSI, including with MRSA^34,35^. Our data provide additional support for cefazolin use as a perioperative antibiotic as sub-MIC treatment did not demonstrate increased bacterial growth. Concerns for penicillin allergy and cross reactivity can lead surgeons to select other options including vancomycin^33^. Multiple studies have demonstrated that cefazolin is safe and effective when a distant history of penicillin allergy is reported^36^. Over time, many patients no longer suffer a penicillin allergy and are skin test negative within a 10 year period^37^. When a self-reported allergic response is recent, antibiotic allergy testing is recommended^38^.

In the case of MRSA infection, vancomycin is the antibiotic of choice; however this can be difficult to appropriately dose based on bioavailability and poor penetration into musculoskeletal tissues^24,39^ and has been shown to have severe side effects from intravenous dosing including nephrotoxicity^40–47^. In 2020, the American Society of Health-System Pharmacists, the Infectious Disease Society of America, the Pediatric Infectious Diseases Society and the Society of Infectious Disease Pharmacists collaboratively worked together in order to create new consensus guidelines for therapeutic monitoring of vancomycin for severe MRSA infections^48^. Prior to these newer recommendations, vancomycin trough levels were considered surrogate markers for measuring the area under the curve (AUC), with an AUC goal of ≥ 400 mcg h/ml deemed sufficient to achieve a clinical therapeutic level of vancomycin based on done and duration. The newer guidelines however no longer recommend using vancomycin troughs as surrogates for AUC, based on a meta-analysis of 14 observational cohort studies that showed vancomycin troughs ≥ 15 were not associated with improved outcomes^49^. In addition, a subsequent meta-analysis of 15 studies assessing the impact of vancomycin trough concentrations on vancomycin induced nephrotoxicity found that there was an approximately 300% increased risk of nephrotoxicity when troughs were at or greater than 15 mcg/ml^44^. Given these findings, as well as a recent meta-analysis study that showed a 32% decrease in risk when AUC-guided dosing was employed over standard trough monitoring for vancomycin^50^, the current guidelines suggest AUC-guided vancomycin dosing over trough monitoring, with AUC goals of 400–600 mcg h/ml. These guideline changes aim to improve overall patient safety, particularly by lowering the risk of vancomycin induced nephrotoxicity, while simultaneously providing more consistent efficacy in the treatment of serious MRSA infections.

The present study is not without limitations. For our SSI model, animals were dosed prophylactically with sub-MIC vancomycin 1-h prior to bacterial inoculation. This is within the SCIP guidelines for perioperative administration, but vancomycin and fluoroquinolones can be given up to 120 min prior to inoculation^3^. In addition, no vancomycin MIC dose was tested for comparison as well as different antibiotic dosing routes. This was not completed as MIC dosing and higher values would be considered appropriate dosing.

## Conclusion

Finally, there is not a direct correlation between in vitro levels and in vivo levels. microbe dynamics, (bacterial load, genetics, persistence, contamination of the wound, implanted material, etc.) and antibiotic dynamics. Here, in vitro dosing was below the MIC and the clinical therapeutic level was below the AUC in our in vivo models.

Our investigation demonstrates the importance of proper antibiotic selection and dosing. Inappropriate dosing of vancomycin resulted in greater bacterial burden, disease severity, and higher infection rates. These results highlight the importance of proper antibiotic stewardship by achieving the needed clinical therapeutic antibiotic levels with appropriate and perioperative dosing to prevent bacterial infections.

## Data Availability

The data generated and analyzed during this study may be available from the corresponding author on a reasonable request to the corresponding author or the corresponding author’s delegate.
